# Cases in Practice, with Remarks upon the Use of Veratrum Viride

**Published:** 1871-02

**Authors:** B. O. Reynolds

**Affiliations:** Geneva, Wis.


### Chicago Medical Journal.

A MONTHLY RECORD OF

*Medicine, Surgery, and the Collateral Sciences.*

EDITED BY J. ADAMS ALLEN, M.D., LL.D.; AND WALTER HAY, M.D.

VOL. XXVIII.—FEBRUARY, 1871.—No. 2.

### Original Communications.

ARTICLE I.—*Cases in Practice, with Remarks upon the Use of Veratrum Viride.* By B. O. REYNOLDS, M.D., Geneva, Wis.

CASE 1. Called on the evening of Sept. 3rd, 1859, to visit William O——, aged about 48, residing near Elk Horn, Wis.; a person of delicate build and constitution.

Was informed that three days before, he was attacked with severe vomiting and diarrhœa, but they had not considered him dangerously ill until to-day.

Found him somewhat delirious, with high fever, and stomach and bowels considerably distended, tympanitic, and tender on pressure. Tongue thickly furred; respiration hurried; pulse, 130. As my notes are silent as to treatment at this visit, I will not, from memory, attempt to give it.

Sept. 4, 9 o'clock, A. M. All the symptoms of last evening greatly intensified, except the febrile heat, which had materially abated; stomach and bowels greatly distended, making no complaint on firm pressure; lungs resonant over their whole extent; indeed, exaggerated, or vesiculo-tympanitic over lower antero-portion; delirious—could scarcely be rallied to respond to questions; great jactitation; pupils moderately dilated, and immovable; tongue flattened, motionless, and covered with a dirty, ash-colored coating, pale at the tip and edges, moist throughout; respirations 65 per m.; pulse scarcely perceptible at the wrist, but by listening to heart found to be 187 per m.

With the assistance of a neighbor, his head was raised, and he was forced to swallow m. x Nor. Tr. Veratrum Viride, mixed with a teaspoonful of water; directing m. viij to be repeated every three hours if he could be made to swallow; continue fomentation to bowels (which I had ordered last evening) and bottles of warm water to be placed to his feet. I left, never again expecting to see Mr. O—— alive.

Visited Mr. O—— again at 8 o'clock, P. M. Found him apparently dying; bathed in perspiration; countenance very pale; skin cool; respirations slow, (12 per m.) but full; with hiccough about once per minute, that seemed to agitate his whole frame, and even shake the bed; pulse 52, full, soft, and uniform; bowels much less enlarged, and much softened. His intellect seemed lucid, but he was unable to speak, or even raise his hand.

On inquiry, I learned they had given m. xx of Veratrum every three hours, instead of eight drops as directed—had given two doses, but said they would give him no more as they believed him dying; also, learned he had vomited freely; had an involuntary movement of bowels and bladder.

I did not inform them of their mistake, as the case was so forbidding at the beginning of the treatment that a fatal termination was liable at any moment, but mentally considered that I now had a well marked case of poisoning by Veratrum Viride to deal with. ℞. Brandy toddy, with capsicum, every fifteen minutes. He had taken but two or three doses when he showed perceptible signs of improvement, when the intervals of giving the stimulus were lengthened. In one hour the pulse had risen to 70; at 10 o'clock, pulse 75; general appearance much improved, at which time he was enabled to drink a little broth. In one hour more the pulse had risen to 75, full and uniform; hiccoughing only at intervals of ten or fifteen minutes.

At 11 o'clock the hiccoughing had entirely ceased; pulse 75; respiration nearly natural; enlargement of bowels diminishing. He continued to improve in every respect up to 1 o'clock, when I

left him after leaving a few Pulv. containing Quin. gr. i, Opi. gr. ss.—one to be taken every four hours—with some general directions relative to his food and comfort.

Sept. 5th, 5 o'clock, P. M. Patient very comfortable, says he feels well; enlargement of stomach and bowels nearly gone, but slightly sensitive to firm pressure; pulse 83; respiration natural; tongue cleaning, and everything indicating a speedy convalescence. ℞. Quinine, gr. ij, every six hours, and Tr. Chlorid. Ferri, m. x, morning, noon and night. Left, with instructions to inform me if patient became worse. In the course of a week I saw Mr. O—— in the streets of Elk Horn looking as well as usual, and he said he felt well.

CASE 2. Called Sept. 11th, 1869, by telegram, to Delavan, Wis., a distance of twelve miles, to see young P——, a lad aged about 17, arriving about 9 o'clock, P. M. Found him under the care of Dr. B. Devendorf, a very intelligent physician of Delavan. Young P—— had been about one week sick with inflammation of bowels. Condition of patient: pulse 140 per m.; respiration (not counted) considerably hurried; tongue thickly furred, very red at tip and edges; bowels greatly enlarged and extremely sensitive to pressure, even of the bed clothes; frequent vomiting; complaining of great pain in bowels, though he was moderately under the influence of morphia. Had recently had free discharges from bowels; renal secretions, scanty and high colored; constant thirst and great febrile excitement.

If my memory serves me, the treatment he had been under for the past twenty-four hours, was about the following, viz.: Calomel, Morphia, Aconite, Digitalis, and thorough fomentations to bowels.

All these in good honest allopathic measure, and yet, as the Doctor expressed it, he "feared he might slip through his fingers."

The results of our deliberation were: That the Morphia, Aconite, and Digitalis, be discontinued; continue alterative doses of Calomel, and the fomentations; also, Nor. Tr. Veratrum Viride, m. iv, every two hours.

In one hour, the pulse had fallen ten beats; in two hours, twenty; in three hours, thirty-five; meanwhile the pain had nearly or quite left the bowels, which had become considerably diminished in size, and less tense; respiration less hurried; skin becoming moist, and had a moderate evacuation of bladder.

At about this period, friends (having been telegraphed to, at Milwaukee) arrived, bringing with them a disciple of the insane Hahnemann, who immediately advised Tr. Aconite, m. 1-8, and a small spoogen of Apis mel (don't recollect the exact amount); but in some way the idea got through his cranium that the patient could take none of these while we were in attendance.

We modestly intimated that we were in the case "for keeps," or until the friends of young P—— desired a radical change of medical attendance.

The friends very justly concluded "that inasmuch as the patient was doing well, and momentarily getting better, no change was advisable."

To this the professional gentleman acquiesced with apparent good grace, yet very modestly and politely insisted upon making an application to patient's bowels, with a pocket homœopathic battery, which he very forcibly and lucidly demonstrated *could do no harm*, while it might possibly do a power of good. But this very harmless request (to the shame of my venerable friend first in attendance be it said) was also refused.

Continued the Veratrum in smaller doses and at less frequent intervals, until about two o'clock, the pulse meantime getting down to 90, and the young man's symptoms improved in every respect. Dr. D—— and the writer, after giving directions for the night, or rather morning, retired.

Sept. 12th, 6 o'clock, A. M. Found patient much improved in every respect. Had rested well, and slept considerable the latter part of the night; perspiring quite freely; has not vomited; bowels much less tender and swollen; tongue looking better; pulse 85. As Dr. D—— had not come round yet, left him a hurried note, and returned home.

Sept. 13th. Received a line from Dr. D—— in which he says, "Patient doing finely." In a few days I met Dr. D——, when he informed me that our patient had a rapid recovery.

CASE 3. Oct. 30th, 1870. Called to see Wallace H—— of Geneva, a lad of 13 years, not remarkably robust when in his best condition. Found him with well marked symptoms of typhoid fever. His mother stated that he had been complaining for more than a week. It is not my design to enter into the minutia of the progress or treatment of this case, during the first few days of its continuance; but I would simply say, that for typhoid fever, it assumed a mild, though unambiguous form, and continued to progress favorably until Nov. 8th, 9 o'clock; on my arrival, the mother stated that W—— had passed a worse night than any since his illness.

At this time he was taking small doses of Quinine, Aconite and Terebinthinate Emulsion. These had been administered for several days, during which time his tongue had begun to clean; bowels becoming less tender; alvine evacuations lessened in frequency and much improved in appearance and consistency. Indeed, up to this time the patient was considered to be doing as well as could be expected.

I find him with tongue nearly clean, but preternaturally red, of a glossy smoothness, with thinly scattered sharp papillæ elevating the mucous membrane, moderately moist, but the mother stated was disposed to become dry during sleep, and when going sometime without drink. Said she had not noticed any diminution in renal secretion; had perspired profusely during the night; had an evacuation of bowels, which she thought improved in appearance. On examination, I found his pulse 113, (they were 75 when I visited him yesterday); slight subsultus tendinum; bowels moderately sensitive to firm pressure, but much less so than during the first week of his illness; mind lucid when thoroughly awake, but disposed to wander when partially asleep; pupils natural, but the eyes had an undefinable quizzical expression, which impressed me unfavorably; lungs resonant; uniform warmth of extremities. Says his head does not ache; complains of no pain.

℞. Suspend Quinia and Aconite; continue T. Emulsion and give Spirits Nit. Ether and decoction of Valerian. Informed the parents that I did not like the aspects of the case at this stage of the disease, and would see him again in the evening.

Nov. 8th, 6 o'clock, P. M. Found patient decidedly worse, had passed a very restless day; pulse 140; respiration rapid; tongue very red, smooth and dry; countenance anxious and hippocratic; delirious—rehearsing his boyish pastimes, etc., picking at the bed-clothes and reaching at imaginary objects—would answer mechanically when sharply rallied—would protrude his tongue with difficulty; great jactitation, extreme restlessness, etc. ℞. N. Tr. Veratrum Viride, m. iv, every three hours. If nausea or vomiting occurred, omit Veratrum and give whisky sling.

Nov. 9th. Nine o'clock, A. M. Patient very much better; had taken medicine regularly; had not vomited; had rested well all night; skin moist; had discharged urine freely; no discharge from bowels; tongue moist, but yet red and smooth; pulse 70, full and soft. Parents very happy, and boy, as he expressed himself, "first rate." ℞. V. Viride, m. iij, four hours; Quinine, gr. i, six hours, with plenty of nourishment.

Evening, six o'clock, P. M. Still improving; takes nourishment with a good relish; pulse 68, full and steady; tongue moist and slightly furred; in a gentle perspiration; bowels less tender; no discharges from them; passes urine freely. ℞. Omit Veratrum, continue Quinine.

Nov. 10th, 9 o'clock, A. M. Still improving; pulse 80. ℞. Continue Quinine; Veratrum Viride, m. iij, morning and evening.

Nov. 11th, ten o'clock, A. M. Patient doing well; pulse 75; tongue beginning to clean. ℞. Cont. Quinine, and give Ferro. P. & W.'s Elix. of Calisaya, ʒ j, morning, noon and night. No medicine during the night.

This case continued to improve steadily, and on the 17th Nov. was discharged, with instructions to continue the ferruginous preparation for a few days.

CASE 4. Dec. 13th, 1870, three o'clock, P. M. Called to see Ida G——, of Geneva. Dr. H. W. Boyce in attendance. The patient, a lively, rosy little sprite when in health, about eleven years old. Scores of times during the past summer and fall, I have observed her dancingly tripping by my office, a perfect picture of health and happiness. She is well fed, clothed and housed, the pet and darling of the family, and cared for with most anxious solicitude by her intelligent parents.

I learned that she had been attacked, about three weeks previous, with scarlatina simplex, and had been constantly under the supervision of Dr. B. since that time; that the eruption came out promptly and thoroughly; that no complications were attendant; that in fact the Dr. and friends considered her to be doing well until yesterday (12th) when she appeared somewhat delirious and had two spasms closely following each other, and to-day has had several more. The Dr. was giving Cal. and Digitalis; had fomented the bowels, bathed the loins with Spts. Terebinth., and had given several injections of Sulph. Magnesia. Present condition: slight puffiness of the face and hands; upper eyelids considerably swollen; bowels moderately enlarged; urine scanty; extremities cold; face flushed; tongue moist, but covered with a dirty, drab-colored coating; pupils slightly dilated, but contracted on exposure to light; eyelids firmly closed and would not open them; a dry, spasmodic cough; partially delirious, talking constantly; complaining of feeling very tired; had vomited some to-day; lungs resonant; pulse 110; respirations 40; great restlessness, constantly tossing about in bed, etc.

*Diagnosis: Uræmic Poisoning.*

It was decided that plenty of water should be given her to drink, and Decoct. Bac. Juniper and Fol. Buchu administered every two hours. As the Dr. was of the opinion that she could be made to swallow but little, small injections of strong solution of Pot. Chloras were to be used every three hours; continue fomentations.

Dec. 14th, eight o'clock, P. M. Requested to see Ida again. Found her to all appearance in a dying state. Dr. Boyce in attendance; had been constantly with her since my last visit. Patient has had several convulsions, but the Dr. thinks they have been partially prevented by free inhalations of Sulph. Ether and Fl. Ext. Valerian. Had kept up injections of Pot. Chloras, had also given one or more spoonfulls of chicken tea, but was unable to get her to swallow but one teaspoonfull of the decoction prescribed yesterday. Said the patient was dying, and he was worn out with fatigue and loss of sleep, requesting me to do whatever I thought advisable. He left the patient in my hands.

I now examined our little patient, whom I found pulseless at the wrist; extremities cold, shrunken and livid; pupils moderately dilated and immovable; constantly tossing in all directions, calling for mother and friends, who were gathered around her couch, momentarily expecting to see her stilled in death; heart beating too frequent to be counted with the ear to the chest, but it aproximated 260 per m.; respirations 95 per m. Impressed with the idea that she could not be made to swallow, and might die in its attempt, I prepared a two-ounce solution of Permanganate Potassa, in which was put Nor. Tr. Veratrum Viride, m. xv, and had it injected per anum. This was immediately rejected. Calling for a teaspoon, and filling it about half full of cold water, I dropped from my phial, Nor. Tr. Veratrum Viride, m. x, into the spoon, and with the assistance of those in attendance enabled the dying Ida to swallow it to its last drop.

Laying her down and looking at my watch I found it was just nine o'clock. Ida remained (doubtless from physical exhaustion) perfectly passive and motionless for the space of ten or fifteen minutes; nothing was heard in that room but the rapid breathing of the little sufferer, and an occasional stifled wail of anguish from the grief-stricken friends. She soon began, however, to be uneasy, but not to the extent she had been before.

In forty minutes from the time the medicine was given, I could plainly discern an improvement in her breathing. Heart beating evidently slower, but too tumultuous to time correctly. At 10 o'clock nearly all present began to whisper "She seems better." At 11 o'clock she was much improved, and was induced shortly after this to drink cold water. At 11½ o'clock counted pulse at carotid, found them 200 per m. At this time for the first, could feel a perceptible thrill at radial artery, but too delicate and unsteady to be counted correctly.

At 12 o'clock finding the pulse but little more reduced in frequency, I gave Ver. V., m. vij, followed by a draught of cold water. At one o'clock, with care the pulse could be counted at the wrist 185. By this time she had become comparatively comfortable. At two o'clock the pulse had lessened in frequency to 145, considerable volume, uniform and steady; respiration and general appearance much improved; extremities warm, and skin moist. Would drink water, but it required considerable firmness and tact to enable her to do it. Three o'clock, pulse 142.

℞. Tr. V. Viride, m. v. At 4 o'clock, her pulse had fallen to 132, increasing in volume as it diminished in frequency; respirations counted, and found to be 44; patient very comfortable; gently perspiring. No appearance of nausea or vomiting. After giving instructions to administer V. Viride, m. iv, every three hours, with abundance of water as often as she could be induced to drink, and to give milk or animal broths occasionally, I left, feeling conscious that we had accomplished a good night's labor.

Dec. 15th, ten o'clock, A. M. Found Dr. B—— in attendance, who expressed great astonishment that Ida was yet alive. Patient much improved, pulse 102; respiration 40; sweating freely; had passed urine; had drank water quite freely. ℞. Tr. V. Viride, m. iij, every four hours. Light to be excluded from room as much as possible. All visitors and superfluous attendants to be excluded from the room, and a liberal amount of water, milk and animal broths to be given her.

Dec. 15, 7 o'clock, P. M. Visited Ida again. Has been very comfortable all day; has taken her medicine regularly; has no nausea; has taken milk and other nourishing drinks; has passed urine freely, also had a small discharge from bowels; countenance calm but somewhat pale; skin cool and covered with moisture; puffiness gone from eyelids; tumefaction of bowels disappearing; intellect clear, and for the first time since I had been visiting her she opened her eyes, and protruded her tongue on my requesting her to do so. I find her pulse 62, soft and full; respiration 33; perspiring freely; extremities warm. There has been no vomiting. Directions: discontinue medicines during the night; keep her very quiet, give plenty of bland nourishment.

Dec. 16, 8 o'clock, A. M. Patient continues to improve; rested well most of the night. Called for coffee and cake, her accustomed breakfast when in health; partook sparingly of weak coffee and cracker. Respiration nearly natural; pulse 95; skin moist; urinary secretion fair. ℞. Quin. gr. i, every six hours; Tr. Veratrum, m. iij, as often as necessary to keep the pulse below 90; also, give Acetate Ammonia, one teaspoonful every four hours.

Nine o'clock, P. M. Patient still improving; pulse 90; respiration good; bowels had moved; considered the patient out of danger. Left her in the hands of Dr. Boyce, with advice to continue tonics in small doses, and control excessive action of heart with Veratrum if necessary

I have heard from Ida daily, and she has continued to improve steadily, and at this writing, Dec. 26th, is almost completely restored.

REMARKS.

As this report has become extended to a much greater length than was at first anticipated, I will not enter upon the discussion of the mooted question, "Is Veratrum Viride a poison?" nor speculate on the modus operandi of its action on the animal economy.

Suppose it is "a virulent poison," as some of our old authors have it (which, however, I do not believe), must the profession ignore its discriminate use on that account? Taking this view of the remedy under consideration, does it not rather lead to the conclusion that in it we have a potent agent, capable, under proper and well chosen circumstances, of accomplishing a vast amount of benefit in the treatment of disease?

If the word "poison" has such alarming import, why not in practice discard the use of Alcohol, Arsenic, Aconite, Ammonia, Antimony, Belladonna, Bismuth, Camphor, Cantharides, Colchicum, Chloroform, Digitalis, Ergot, Ether, Hyosciamus, Hellebore, Henbane, Hemlock, Iodine, Lead, Lactucarium, Lobelia, Mercury, Nux Vomica, Opium, Stramonium? The entire class of mineral, and some of the vegetable, acids, together with many other articles in daily use with the profession—even Epsom Salts and Cream Tartar—would belong in the same category, (*Vide* Taylor on Poisons, pp. 19, 305), for they have been known to destroy life, which fact, I think, cannot be substantiated in regard to the article under consideration.

In the use of Veratrum in Case 1, I was at the time as ready to censure as to praise. That Mr. O—— would have died in a few hours without its use, I was satisfied. That its over-dose came very near killing him, I felt also positive; and that it ultimately saved his life, I am, and ever shall be, morally certain.

Case 2 having been judiciously treated from the beginning, might possibly have recovered without its use. But I ask, what remedy or remedies have we, that would have caused so perceptible a change for the better in so short a time?

In a continuous practice of twenty-four years, I have treated many cases of this grave disease, both in army and private practice, and I here affirm that I never saw a case of this disease make half such rapid progress towards health, as was exhibited in this case under the use of Veratrum Viride.

In Case 3, it seemed "like a strong man armed" to lift little Wallace from the declivity which leads to death, and rapidly restored him to health and happiness.

In Case 4, its workings were more like magic than anything real. It seemed to clutch, as it were, this child from the icy embrace of death, and but for its timely use, I have no doubt that dear Ida would have been "where the woodbine twineth," within a few short hours from my arrival on that eventful evening.

